# The Value of Fecal Markers in Predicting Relapse in Inflammatory Bowel Diseases

**DOI:** 10.3389/fped.2017.00292

**Published:** 2018-01-19

**Authors:** Bianca J. Galgut, Daniel A. Lemberg, Andrew S. Day, Steven T. Leach

**Affiliations:** ^1^School of Women’s and Children’s Health, University of New South Wales, Sydney, NSW, Australia; ^2^Department of Gastroenterology, Sydney Children’s Hospital Randwick, Sydney, NSW, Australia; ^3^Department of Paediatrics, University of Otago, Christchurch, New Zealand

**Keywords:** inflammatory bowel diseases, prognosis, fecal marker, calprotectin, lactoferrin, S100A12

## Abstract

The inflammatory bowel diseases (IBDs) are lifelong chronic illnesses that place an immense burden on patients. The primary aim of therapy is to reduce disease burden and prevent relapse. However, the occurrence of relapses is often unpredictable. Current disease monitoring is primarily by way of clinical indices, with relapses often only recognized once the inflammatory episode is established with subsequent symptoms and gut damage. The window between initial upregulation of the inflammatory response and the recognition of symptoms may provide an opportunity to prevent the relapse and associated morbidity. This review will describe the existing literature surrounding predictive indicators of relapse of IBD with a specific focus on fecal biomarkers. Fecal biomarkers offer promise as a convenient, non-invasive, low cost option for disease monitoring that is predictive of subsequent relapse. To exploit the potential of fecal biomarkers in this role, further research is now required. This research needs to assess multiple fecal markers in context with demographics, disease phenotype, genetics, and intestinal microbiome composition, to build disease behavior models that can provide the clinician with sufficient confidence to intervene and change the long-term disease course.

## Introduction

The inflammatory bowel diseases (IBD) are lifelong, relapsing-remitting diseases affecting physical, psychological, familial, and social aspects of life, and encompass the two main subsets, Crohn’s disease (CD) and ulcerative colitis (UC). As the timing of relapse is unpredictable, and current monitoring is symptoms-based, there remains a window between the initial upregulation of the inflammatory response and the onset of clinical symptoms at which point the inflammatory episode is well established. A possibility therefore exists to identify patients in this window and prevent the clinical relapse. Furthermore, this may help define the future risk of relapse, aid treatment decisions assess response to treatment, and evaluate therapeutic goals such as mucosal healing (MH).

One method to assess this risk may be endoscopy, which allows direct evaluation of mucosal lesions, where the severity of these lesions and can be used to assess MH. In IBD patients undergoing treatment, MH is associated with lower relapse rates (1, 2). CD patients who achieved MH with infliximab had longer relapse-free intervals than those failing to achieve full healing (3, 4). However, endoscopy is invasive, labor intensive, and can only assess the gastrointestinal mucosa. Furthermore, there are risks associated with endoscopy, the most serious of which is risk of colonic perforation which can be as high as 0.3% (5). Therefore, the use of endoscopy as means of predicting relapse is not suitable for regular use.

Additional imaging techniques including radiography, magnetic resonance enterography, wireless endoscopy, and computed tomography can also provide complimentary evidence in diagnosis, prognosis, and disease monitoring (6). In this sense, the utility of imaging IBD patients can provide additional information to endoscopic mucosal evaluation and can describe the mucosal wall and identify stricturing and penetrating complications. However, each method also has disadvantages which can include radiation exposure, cost, and time.

Although method of visualization allows for the direct assessment of intestinal inflammation, non-invasive, accurate, and acceptable tests are needed as a more practical prognostic method. In this respect, disease biomarkers may provide the best method for predicting IBD prognosis. Their roles in diagnosis and assessment of disease activity have been well documented. This review aims to provide a summary of current knowledge of the use of fecal biomarkers in their capacity to aid prognosis in IBD.

## Biomarkers

Laboratory markers have been investigated as a means of reducing the need for endoscopic assessment by providing an accurate, objective measurement of inflammation (7). Studies have found that biomarkers such as fecal calprotectin (FC) may have a role in monitoring for post-resection recurrence, particularly of CD (8, 9). While the utilization of biomarkers instead of endoscopic monitoring is important in this setting, the focus of this paper is on predicting relapse outside of post-surgical recurrence.

### Fecal Markers

The current assessment of intestinal inflammation primarily consists of clinical measures, radiology, endoscopy, histology, and serum markers. However, there is gaining acceptance in this process for fecal markers, such as calprotectin, lactoferrin, and S100A12 (10). Novel markers, such as high-mobility group box 1 (HMGB1) protein, are also being investigated (11, 12).

#### Calprotectin

Calprotectin is a 36 kiloDalton, calcium- and zinc-binding protein that comprises up to 60% of cytosolic proteins in neutrophils, being released during apoptosis or necrosis (13, 14). It contributes to inflammatory process regulation and has antibacterial, antifungal, and antiproliferative properties (14). Its fecal concentration is therefore proportional to neutrophilic influx into the intestinal tract, which is a feature of active IBD (7). After excretion, FC remains stable in the feces for 1 week at room temperature (14). However, its considerable daily variation in those without intestinal inflammation or neoplasia suggests that factors discrete from inflammatory disease may affect FC levels (15).

The potential role of FC in IBD in predicating the risk of relapse has been well investigated with the key studies summarized in Table 1. An initial study be Tibble et al. (16) studied 80 IBD patients in clinical remission for 1–4 months (43 CD, 37 UC), measuring FC at baseline. The median FC levels in the relapse group (CD 122 mg/L, UC 123 mg/L) were significantly higher than the non-relapse group (CD 41.5 mg/L, UC 29.0 mg/L, *P* < 0.0001 for CD, UC, and all patients). The authors used an FC cutoff level of 50 mg/L (normal levels < 10 mg/L), with patients above this cutoff having a 13-fold increased risk of relapse within 12 months. This cutoff gave a sensitivity and specificity of 90 and 83%, respectively, in predicting relapse within 1 year. Subsequently, Costa and colleagues (17) conducted a similar study to determine whether the predictive value of calprotectin differed between CD and UC. The authors found a non-significant twofold increased relapse risk in CD and a significant 14-fold increase in UC, for those in clinical remission with FC > 150 μg/g. These data strongly suggested that FC was a superior predictive marker in UC than CD. However, the specificity value provided by Tibble and colleagues was for all IBD, not CD alone. As such, the comparison with Costa’s results must be made with some reservation.

**Table 1:** Fecal calprotectin in predicting inflammatory bowel disease (IBD) relapse.

Reference	Cohort	Sensitivity (%)	Specificity (%)	Cutoff	Remission duration at entry (month)	Study end point
Tibble et al. (16)	80 (IBD)	90	83	50 mg/L	1–4	12

Costa et al. (17)	38 (CD)	87	43	150 μg/g	1–12	12
	41 (UC)	89	82			

D’Inca et al. (23)	65 (CD)	65	62	130 mg/kg	3–36	
	97 (UC)	70	70			

Gisbert et al. (24)	163 (IBD)	100	70	167 μg/g	6	3
		69	75			12
	89 (CD)	69	76	169 μg/g		12
	74 (UC)	69	74	164 μg/g		12

Kallel et al. (26)	53 (CD)	80	91	340 μg/g	>3	12

Garcia-Sanchez et al. (25)	135 (IBD)	80	60	120 μg/g	>3	12

Sipponen and Kolho (28)	72 (IBD)	32	80	82 μg/g	>3	12
		38	72	108.5 μg/g		
		83	24	1,005 μg/g		

Naismith et al. (27)	92 (CD)	80	75	240 μg/g	Not specified	12

*CD, Crohn’s disease; UC, ulcerative colitis*.

In response to these previous studies, Pardi and Sandborn (18), postulated that the differing remission times prior to inclusion in the study could contribute to the specificity discrepancy—the Tibble study recruited patients who were in remission for 1–4 months, whereas the Costa study recruited those in remission for 1–12 months. The authors suggested that the predictive value of calprotectin may decrease with increased remission times.

A further variable to consider is the association of FC normalization with MH (19). More than half of UC clinical remission cases are associated with endoscopic and histological normalization (20), whereas in CD the proportion is only 10% (21). Therefore, a higher proportion of UC cases will have normalized mucosa, and hence normalized FC levels, and an elevated FC would likely have increased predictive accuracy when compared to CD.

Hanaway and Roseth (22) provide more commentary on the non-equivalency of the two patient populations. The Costa study had a higher proportion of CD patients with ileal CD (71%) compared to those with ileocolitis or colitis (29%). In the Tibble study, however, these values were 47 and 53%, respectively. This suggests that FC is a poor predictor of relapse in ileal CD and differences in the percentage of ileal patients in the study cohorts are likely to impact the overall findings (22).

In a further study, D’Inca et al. (23) assessed 162 patients (65 CD, 97 UC) in clinical remission for 3–36 months. The authors found a subgroup of colonic CD had significantly higher levels of FC in the relapsers than the non-relapsers [176.7 mg/kg (95% CI = 151–203) versus 75.1 mg/kg (95% CI = 21–129; *P* = 0.041)], whereas this difference was insignificant for the overall CD cohort. This further suggests the inaccuracy of FC as a predictor of relapse in ileal CD.

Gisbert et al. (24) conducted a similar study, with 163 patients (89 CD, 74 UC) in clinical remission for 6 months providing a stool at baseline with follow-up at 12 months. FC levels were higher in the relapse group than the non-relapse group with relapse risk increasing from 7.8% for those with FC levels below 150 μg/g to 30% for those with FC levels exceeding 150 μg/g (*P* < 0.001). The authors calculated that for a 3-month time frame, the accuracy of FC as a relapse predictive test increased to a sensitivity of 100% and specificity of 70%.

Garcia-Sanchez et al. (25) conducted a subsequent, prospective study with 135 patients (66 CD, 69 UC) who had been in clinical remission for >3 months, and similarly collected baseline stool samples and followed up at 1 year. They found the sensitivity and specificity of predicting relapse for colonic CD to be 80 and 60%, respectively, which was markedly higher than for ileal CD.

In a CD-specific study, Kallel et al. (26) recruited 53 patients in clinical remission, collecting stool at baseline with 1 year follow-up. Median FC in the relapse group was significantly higher than the non-relapse group with an elevated FC level giving an 18-fold increased relapse risk (*P* < 0.001). In this study the authors used a cutoff of 340 μg/g and the higher relative risk compared to other studies, and it is most likely attributable to the higher cutoff value used. The authors also excluded CD patients with only small bowel disease, which may have also increased accuracy, despite the relatively small sample size. Another difference was that the majority of patients were receiving azathioprine therapy. This suggests the population may have had more severe disease, higher levels of FC, and a higher likelihood of relapse compared to previous studies. Nevertheless, studies with similar patient populations have also shown the good predictive utility of FC (27).

In contrast, a study by Sipponen and Kolho (28) reported that FC was not elevated in their relapse group compared to those that did not relapse. The study was in a pediatric cohort including CD, UC, and IBD unclassified. Therefore, there is the potential for the predictive utility of FC to differ from adult to childhood disease.

A meta-analysis by Mao et al. (29) of six prospective studies included 672 IBD patients (354 CD, 318 UC). The pooled sensitivity and specificity of FC predicting relapse were 78% (95% CI = 72–83%), and 73% (95% CI = 68–77%). Of all enrolled CD patients, FC predictability was better for ileocolonic and colonic, rather than ileal CD. This study reinforces previous findings that FC as a predictive marker may only be suitable for colonic and not ileal disease.

More recently, there have been several investigations of FC maintaining remission following infliximab maintenance therapy (30, 31). These investigations report long-term remission was associated with low FC levels. It has also been suggested that FC measurement may be more informative if recorded on a continuous scale with serial measurements, as opposed to being a dichotomous variable with a single cutoff point (32).

Overall, there is a consistent finding for a potential role for FC in predicting relapse in IBD. However, the sensitivity and specificity vary considerably between studies even when there is a consistent end point of 12 months in predicting relapse. A possible means of increasing accuracy may be to reduce this end point, as the Gisbert study explored. Additionally, it has been shown that FC is a better predictor of relapse in colonic CD than ileal CD. Future research should consider stratifying the CD cohort to determine the predictive value of FC in these subgroups.

#### Lactoferrin

Lactoferrin is an iron-binding glycoprotein of secondary granules in neutrophils. It is a primary factor in the acute inflammatory response, being released during degranulation and neutrophil adhesion to vascular endothelium (33). As such, fecal levels of lactoferrin rise quickly with neutrophilic influx during inflammation, and it has been shown that this is a highly sensitive marker for fecal neutrophil infiltration (34). It is less stable than calprotectin, resisting degradation for 5 days at room temperature, but is unaffected by multiple freeze/thaw cycles (34, 35).

The Gisbert study (24) also measured lactoferrin in their study cohort. They found that a positive result for lactoferrin correlated with an increased relapse risk (25% relapse risk with positive result versus 10% with negative result, *P* < 0.05), and predicted relapse with a sensitivity of 62% and specificity of 65%. Analyzing CD and UC separately, the sensitivity and specificity for CD were 77 and 68%, respectively, and for UC these were 46 and 61%. Sensitivity and specificity increased, similar to calprotectin, when only considering the prediction of relapse within the subsequent 3 months (sensitivity 100%, specificity 62%). The limitation of lactoferrin in this study was that it was assessed qualitatively—that is, the result was positive or negative depending on its presence, as defined by a non-standardized cutoff value.

More recently, Yamamoto et al. (36, 37) conducted two studies in adult CD and UC patients, respectively. A cutoff value of 140 μg/g was 67% sensitive and 71% specific in predicting relapse within 12 months the CD cohort, and 67% sensitive and 68% specific in the UC cohort. It is worth noting that the CD study included only 20 patients who were post-ileocolonic resection. More research is needed to evaluate the potential for lactoferrin in predicting IBD relapse, and whether a quantitative assessment would improve predictive power.

#### S100A12

S100A12 is a S100 protein similar to calprotectin, and expressed as a cytoplasmic granule in neutrophils. It has pro-inflammatory properties, most notably potent chemotactic activity, and is upregulated by TNF-α (38, 39). This is important for IBD as it may contribute to leukocytic infiltration, and therefore its measurement may reflect the presence and severity of intestinal inflammation (40). It can be measured in serum or feces and is stable for 7 days after collection (41). Literature exists regarding its role in diagnosis and monitoring disease activity; however, its role in prognosis has not been extensively explored.

Däbritz et al. (42) recently studied 147 adults and 34 children with IBD (61 CD, 120 UC) over a 3-year period. Fecal levels of S100A12 were found to be significantly higher in the relapse group than the non-relapse group, with a baseline level of >0.5 mg/kg being significantly associated with relapse within 18 months. Time course analysis revealed an increase of S100A12 concentrations up to 6 months before clinical relapse. Their cutoff point of 0.43 mg/kg was 70% sensitive and 83% specific for predicting IBD relapse at 8–12 weeks before the relapse episode. This study suggests that S100A12 also has a potential role in predicting relapse but further investigations are needed.

### Novel Markers

There is increasing research into novel fecal markers, such as HMGB1. HMGB1 has been shown to reflect intestinal inflammation in mouse models, correlating significantly with endoscopic indices (SES-CD, endoscopic Mayo subscore) but not with disease activity indices (CDAI, partial Mayo score) (12). The same research group measured HMGB1 and FC in 204 human IBD patients (11). They found that fecal HMGB1 expression was significantly raised in pediatric and adult IBD patients when compared with age-matched controls and strongly correlated with disease severity. Additionally, they found that HMGB1 correlated with FC in these patients and that only fecal HMGB1 identified histologic inflammation in patients with clinical and endoscopic remission. As such, it may prove to be a robust marker for subclinical intestinal inflammation. In addition, fecal immunochemical test (FIT), primarily utilized as a bowel cancer screening, has shown to be useful in prediction of relapse in IBD (43). Importantly has also shown to provide distinct information from FC with regards to relapse prediction to indicate that a combination of FC and FIT may increase predictability (44).

## A View to the Future

There has been an evolution of the therapeutic approach to treating IBD from managing symptoms to the current approach of healing. However, even with this change in approach allows, there is still the potential occurrence of relapse which is often unpredictable. Relapse will cause subsequent damage to the gut and poorer long-term disease outcomes. Therefore, accurate and acceptable means of predicting relapse are needed to reduce intestinal damage and potentially change the long-term disease course. Currently available methods of monitoring disease are inadequate to reliably predict a relapse event. However, new disease monitoring methods, such as existing and novel fecal biomarkers, are showing promise as relapse predicative tools.

Fecal calprotectin measurement is now widely available and is being incorporated into routine clinical practice for the diagnosis and monitoring of IBD. Although, it is still limited in fulfilling this role as a prognostic marker in long-term disease course. The limitation of FC is that precise interpretation of an isolated measure remains difficult when it is only moderately elevated. FC provides a sensitive measure of inflammation, with elevated levels preceding a clinically significant relapse by months. But associated with this high sensitivity is poor specificity, as calprotectin can also be moderately elevated in the absence of clinically significant events. Therefore, a single measure of a moderately elevated FC is ambiguous and currently does not provide a clear indication of when clinical intervention is required to change the long-term disease course. This remains the problem of FC in isolation when used for disease monitoring.

Nevertheless, there is a building interest in defining a viable prognostic tool for IBD. The use of biomarkers is only one mechanism, among others, that may be able to provide prognostic information. However, the authors would argue that fecal biomarkers offer a promising solution to this problem. The advantages of fecal biomarkers are that samples (feces) are easy to obtain, can be collected at home, can be serially obtained, and can be relatively easy to analyze with the sample posted to the laboratory for analysis. This allows the patient to regularly monitor their disease without the need to visit the clinician. Therefore, fecal biomarkers offer a convenient, non-invasive, low cost option for disease monitoring. The problem with fecal biomarkers is that, currently, they do not provide the clinician with sufficiently detailed information to confidently act and intervene to change the long-term disease course.

This provides the biggest challenge in the immediate future, to establish how fecal biomarkers may be able to provide sufficiently accurate and reliable prognostic information to allow for preventive therapy to avoid a relapse event and change the disease course. There are multiple options which may increase the accuracy and reliability of fecal biomarkers which include measuring and interpreting multiple fecal markers, interpreting the behavior of fecal markers overtime, and interpreting fecal markers in the context of additional clinical information including demographics, disease phenotype, genetics, and intestinal microbiome composition. Nevertheless, it is likely that no single marker in isolation will be sufficient to accurately provide prognostic information and it is likely that combinations of markers, as well as additional information, will be used to build models to predict disease behavior which will be used to assist in changing the disease course. The challenge will be to balance access, cost, and effort required in gaining this biomarker information against the health outcomes that are achieved.

## Conclusion

The literature surrounding predictive markers for IBD is promising. Fecal biomarkers provide a non-invasive, objective tool for relapse assessment. The research into the predictive value of fecal markers, such as calprotectin, lactoferrin, S100A12, and novel markers like HMGB1, indicate these markers may have a role in predicting relapse. However, it is currently unclear how to use these prognostic indicators to change the disease course. Therefore, further studies are needed to provide additional information such as definitive and appropriate cutoff points and realistic but accurate end points for the relapse period. Nevertheless, it is likely in the future there will be wider use of existing fecal biomarkers, as well as new markers including novel fecal markers, for disease monitoring in IBD. These markers will be used to predict short- and medium-term disease outcomes, including likelihood of relapse. These predicative markers will play a key role in driving a shift in attitude as to how to treat IBD, from the currently held view of therapy to control disease symptoms to therapy to change the disease course.

## Author Contributions

DL, AD, and SL conceived the manuscript, BG undertook the literature review, BG and SL contributed to writing, and DL and AD reviewed and edited the manuscript.

## Conflict of Interest Statement

The authors declare that the research was conducted in the absence of any commercial or financial relationships that could be construed as a potential conflict of interest.
